# Detection of Arctic and European cluster of canine distemper virus in north and center of Iran

**Published:** 2015-09-15

**Authors:** Somayeh Namroodi, Amir Rostami, Keyvan Majidzadeh-Ardebili, Arash Ghalyanchi Langroudi, Abbas Morovvati

**Affiliations:** 1 *Department of Environmental Sciences, Faculty of fisheries and Environmental Sciences, Gorgan University of Agricultural Sciences and Natural Resources, Gorgan, Iran; *; 2 *Department of Internal Medicine, Faculty of Veterinary Medicine, University of Tehran, Tehran, Iran; *; 3 *Tasnim Biotechnology Research Center, Faculty of Medicine, AJA University of Medical Sciences, Tehran, Iran; *; 4 *Department of Microbiology, Faculty of Veterinary Medicine, University of Tehran, Tehran, Iran; *; 5 *Department of Microbiology, Islamic Azad University of Qom Branch, Qom, Iran.*

**Keywords:** Canine distemper virus, Hemagglutinin gene, Nucleoprotein gene, Phylogenetic analysis

## Abstract

Canine distemper virus (CDV) creates a very contagious viral multi-systemic canine distemper (CD) disease that affects most species of Carnivora order. The virus is genetically heterogeneous, particularly in section of the hemagglutinin (H) gene. Sequence analysis of the H gene can be useful to investigate distinction of various lineages related to geographical distribution and CDV molecular epidemiology. Since vaccination program is conducted only in large cities of Iran, CD still remains as one of the major causes of death in dogs in this country. In order to monitor H gene, CDV has been detected in 14 out of 19 sampled dogs through the amplification of nucleoprotein (NP) gene in nested-PCR assay. In the next step 665 bp of H gene was amplified in 9 out of 14 NP-gene positive dogs. Phylogenetic analysis distinguished two distinct CDV genotypes in Iran. JN941238 has been embedded in European cluster and JN941239 has been embedded in Arctic cluster. Nucleic analysis has been shown high difference among both Iranian CDV lineages with CDV vaccine strains.

## Introduction

Incurable canine distemper (CD) is a lethal and contagious disease caused by canine distemper virus (CDV). Members of *Canidae* family are the main reservoirs of CDV, while other species in Carnivora order can also be afflicted.^1^^,^^2^ Canine distemper virus belongs to order Mononegaviral, genus *Morbillivirus *of the family *Paramixoviridae *which is closely related to other Morbilliviuses such as measles virus (MeV), rinderpest virus (RPV) and peste des petits ruminants virus (PPRV).^3^^,^^4^

The negative non-segmented single stranded RNA of CDV is enclosed in a nucleocapsid which is surrounded by lipoprotein membrane.^1^

CDV genome is composed of six genes that produce 8 proteins, two non-structural proteins (C and V), and 6 structural ones: Two membrane glycoproteins (the fusion and the hemagglutinin), matrix protein, nucleocapsid protein, large protein and phosphoprotein.^5^

The most definitive method for diagnosis of this virus is the amplification of its different genes through molecular methods.^1^ Since the nucleoprotein (NP) gene is the most conserved gene of CDV; amplification of the NP gene is the best way to identify various strains of CDV in biological specimens.^6^^-^^9^

H protein is responsible for binding the virus to the host cell. It also stimulates the immune system of the host leading to the protection response against the virus.^10^

The highest level of mutation occurs in H gene. Thus, designing specific primers targeting the H gene make it possible to differentiate the field CDV lineages.^11^

Sequence and phylogenetic analysis of CDV strains in suspected animals revealed that different CDV strains were divided in 6 clusters related to the geographic location of the CDV strains in the world. 

These lineages were named America 1 (consisting of vaccine strains), America 2 (with US isolates from domestic dogs and Canidae), Asia 1, Asia 2, European and Arctic – Like (formed by sequence similar to PDV-2).^6^^,^^11^^,^^12^

There is no molecular data about the CDV lineages circulating in Iran. To elucidate the lineages of CDV which are circulating in Iran, sequence and phylogenetic analysis based on the genetic heterogeneity in the H gene has been performed in this study.

## Materials and Methods

**Sampling.** In this study, samples from 19 unvaccinated rural dogs with clinical signs of CD, such as fever, purulent ocular and nasal discharge, tonsillitis, bronchitis, and gastroenteritis disturbance were surveyed. The samples were obtained from Veterinary Medical Teaching Hospital of University of Tehran (Center of Iran) and local animal hospitals of Golestan province (North-East of Iran) from 2009 to 2014. All the dogs were under12 months of age. History, signalment, clinical findings and vaccination status were recorded in special forms (data not shown). The specimens used in this study included whole blood (n = 18), ocular swab (n = 19), and blood serum (n = 18). In case of dog’s death or euthanasia (n = 3), tissue samples have been collected from various organs (lung, bladder, kidney, intestine and stomach). All the samples have been prepared in phosphated buffered saline (PBS) and frizzed in –70 ˚C before consuming in molecular studies.

**RNA extraction and cDNA synthesis. **RNA extraction kit (Bioneer Co., Daejon, Korea) were used following the manufacturer’s instruction for extraction of RNA from all specimens, Vero cell infected with Onderstepoort vaccine strain of CDV (Ond-CDV, positive control), and also ultra-pure water (Negative control). The extracted RNA was reverse-transcribed into cDNA using tow step reverse transcription polymerase chain reaction (RT-PCR) kit (Vivantis, Selangor, Malaysia) following the manufacturer’s recommended reaction conditions. After cDNA amplification, the product was stored at –20 ˚C until further molecular analysis. 

**PCR and Nested-PCR. **Tree pairs of primers targeted to the CDV NP and H genes were designed using sequences obtained from GenBank and Gene Runner (Version 3.05; Hastings software Inc., Hastings, USA), (Table 1).^13^ Detection of NP gene was performed using two sets of primers (NP1 and NP2) through Nested- PCR assay.

The RT-PCR and Nested-PCR assay were run in 25 μL final volume. For RT-PCR assay, 5 μL cDNA and 17 μL 1X PCR master mix (Vivantis, Selangor, Malaysia) and For Nested-PCR, 20 μL 1X PCR master mix and 1 μL DNA were used. Data of the PCR thermocycler programs is not shown. The Ond-CDV infected Vero cells and ultra-pure water were the samples which have been used in each stage of molecular assays as positive and negative control, respectively. In order to elucidate the sizes of PCR products, electrophoresis on a 1% ethidium bromide agarose gel has been performed using the ultraviolet (UV) imaging and 100 bp ladder that was used as a DNA size marker (Fermentas Co., Berlin, Germany).

**Table 1:** Oligonucleotides used for the amplification of H and NP gene of CDV

**Primer**	**Sequence (5' to 3')**	**Size of product**
**F CDVH gene**	TGAAAGAGGATATGGAGAAATCAG	665 bp
**R CDVH gene **	CAAGGAAGCCAGTGTCAACTC	
**F CDVNP1 gene**	GGGTCGAAAGCTCAAGCAC	777/778 bp
**R CDVNP1 gene**	CTGACACTAGCTGACCTCTTC	
**F CDVNP2 gene**	CCTGCTCGCTAAAGCAGTG	520 bp
**R CDVNP2 gene**	CCCTCCCATGGAGTTTTCA	

F: Forward, R: Reverse, bp: Base pair

**Phylogenic analysis. **For sequencing two obtained CDV H gene fragments (CDV 8, and CDV 10), the PCR products have been extracted from the agarose gel by the gel purification kit (Cat No. K-3034; Bioneer, Daejon, Korea). Purified DNA sequenced bi-directly using forward and reverse primers (Seqlab, Gottingen, Germany). PPartial nucleotide sequences of CDV H gene were aligned with those available in GenBank using the program Align Plus 4 (Scientific and Educational software; Cary, USA) and free clustalX.^14^ CDV sequences were checked by the MEGA 4.0 software (Biodesign, Tempo, USA), and then sequence and phylogenetic analysis performed by this software as well. The phylogenetic tree was created by the neighbor-joining method and distances values with a 1000 bootstrap replicates.^15^

## Results

**Fig. 1:**
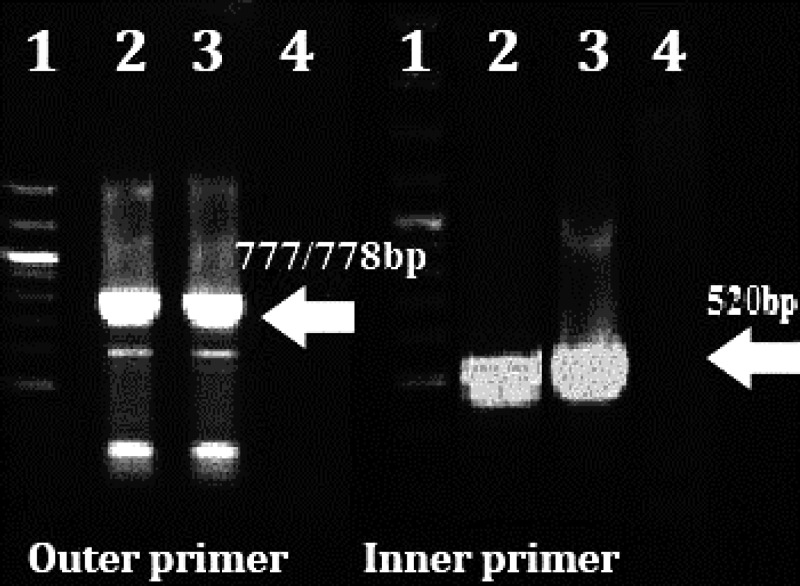
RT-Nested PCR results on agarose gel (1%) electrophoresis of different samples by outer and inner primer of NP gene.  Lane 1) Molecular marker (100-bp ladder); Lane 2) CDV Onder-stepoort strain; Lane 3) Suspected sample; Lane 4) Ultra-pure water. Numbers on the left are molecular sizes (in base pairs).

**Nested PCR and PCR. **The expected 777-778 bp CDV NP gene was identified in three animals and 520 bp pieces were detected in different samples of 14 dogs. In addition, 665 bp of H gene were amplified in 9 out of 14 Nested-PCR positive dogs (Fig. 1).

**Fig. 2:**
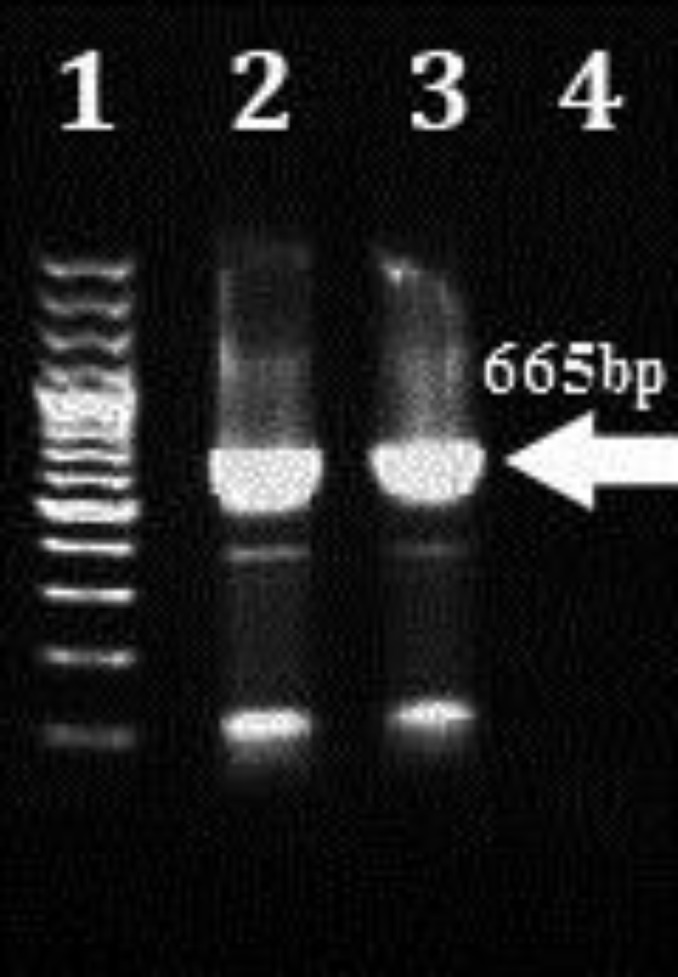
PCR results on agarose gel (1%) electrophoresis of NP gene positive samples by primers of H gene: Lane 1) Molecular marker (100-bp ladder); Lane 2) CDV Onderstepoort strain; Lane 3) Suspected sample; Lane 4) Ultrapure water. Numbers on the left are molecular sizes (in base pairs)

**Sequence and phylogenetic analysis. **The analyzed sequences are available in GenBank data base with accession numbers**: **JN941238 (CDV 8) and JN941239 (CDV 10). Phylogenetic analysis also has been detected two distinct lineages in mentioned regions of Iran. JN941239 and JN941238 have been entered in the Arctic and European cluster, respectively (Fig. 2). 

**Fig. 3:**
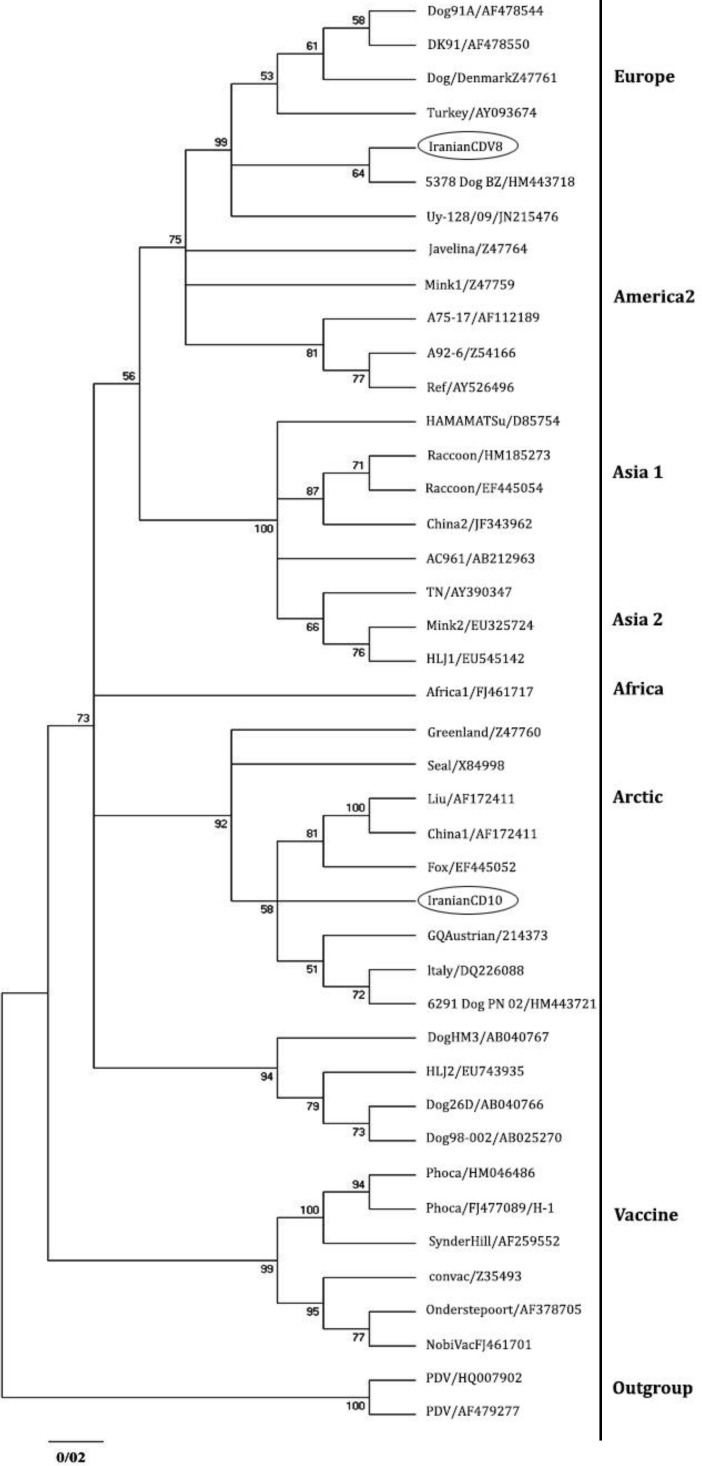
Phylogenetic relationship between different CDV strains based on the nucleotide sequences of the H gene obtained from GeneBnak

Sequence analysis detected 91.48% similarity between two Iranian H sequences. Phylogenetic analysis revealed that two Iranian sequences were obviously different from vaccine strains (Fig. 3). Homology (90.93%) was identified in Iranian H sequences with the America 1 cluster, which is consisting of old vaccine strains. Iranian H sequences were different from 90.11% to 91.99% to Ond-CDV strain which is the classic indicator of this cluster.

## Discussion

Collecting a large set of data related to genetic diversity of CDV is crucial to study the source of the CDV strains and molecular epidemiology. In this study, Unexpectedly, JN941238 (CDV 8) was clustered along with European genotypes and JN941239 (CDV 10) indicated high similarity with Arctic genotypes. Compared to other European sequences considered in this study, JN941238 showed the lowest diversity (1.34%) with HM443718 lineage isolated from a dog by others.^16^

From pronounced connection between these two CDV lineages it can be concluded that HM443718 lineage is possibly the ancestor of Iranian-European lineage (JN941238). On the other hand, the nearest and the only neighboring country of Iran in which the European lineages has been observed is Turkey.^17^ More than 98.00% of similarity between HM443718 and Turkish-European lineage (AY09374) can refer to possibility of transferring or at least correlation of European CDV lineage (JN941238) to Iranian ecosystem through this country.

The arctic viruses were identified in the late 1980s. At that time, these lineages were detected as responsible for the mortality of seals in north west Europe and Siberia.^18^^,^^19^

However, since then, the presence of Arctic-Like lineages are reported in many European and American countries. China is located in Asia where presence of Arctic-like lineages was reported in it. JN941239 (CDV 10) revealed highest correspondence to Italian sequences of the Arctic lineages, DQ226087, that has been distinguished in a dog by Martella *et al*.^11^

Similarity (90.93%) was detected between Iranian H sequences and vaccine strains. The level of genetic variation between these Iranian lineages should be regarded as a possible factor that can lead to resurgence of distemper in vaccinated dog population like other countries where vaccinated dog were affected by field CDV strains.^20^^,^^21^

As it is observed, so far, molecular data have not been documented about CDV genome in the closest countries to Iran, except for Turkey. Hence, it cannot be accurately determined which country is source of transferring these lineages to Iran. Nonetheless, legal or illegal trading of domestic and wild Canidae could lead to emergence of these CDV lineages in Iran. Further studies seem necessary to determine whether the identification of these types of lineages in Iran refer to their permanent establishment in Iran or these results are just an occasional finding. 

In conclusion, it was very lucky to detect two lineages in Iran only by sequencing two H gene sequences in two different geographical areas in a vast country like Iran. However, further studies on CDV genome in different ecological area and susceptible animals in Iran are required to improve the knowledge about host immune system stimulator protein, H protein, with high genetic diversity. Also these kinds of data is required for investigation of the commonest CDV in the world as well as producing more effectual vaccines against CD specific to dogs.
